# Mechanical Properties of Lattice Structures with a Central Cube: Experiments and Simulations

**DOI:** 10.3390/ma17061329

**Published:** 2024-03-14

**Authors:** Shuai Guo, Yuwei Ma, Peng Liu, Yang Chen

**Affiliations:** 1 Department of Mechanical and Electrical Engineering, Guilin University of Electronic Technology, Guilin 541004, China; guoshuai@kyky.com.cn (S.G.); hao122012@163.com (P.L.); 2 Department of Architecture and Transportation Engineering, Guilin University of Electronic Technology, Guilin 541004, China; myw@guet.edu.cn

**Keywords:** lattice structure, energy absorption, additive manufacturing, printing defects, finite element model

## Abstract

In this study, a new structure is proposed based on the body-centered cubic (BCC) lattice structure by adding a cubic truss in the center of the BCC structure and denoting it TLC (truss–lattice–cube). The different dimensions of the central cube can notably affect the mechanical properties of the lattice structure. With a fixed length (15 mm) of a unit cell, the optimal size for the central cube is determined to be 5 mm. Quasi-static compressive tests are performed on specimens made of polylactic acid (PLA) using additive manufacturing technology. The deformation characteristics of the new structure are analyzed in detail by experiments and numerical simulations. Compared to the BCC structure, the mechanical properties of the TLC structure were significantly improved. The initial flow stress of the TLC increased by 122% at a strain of 0.1; the specific strength enhanced by 293% at a strain of 0.5; and the specific energy absorption improved by 312% at a strain of 0.6. Printing defects in the lattice structure may remarkably damage its mechanical properties. In this work, incorporation of microcracks into the finite element model allows the simulation to capture the influence of printing defects and significantly improve the predictive accuracy of the simulation.

## 1. Introduction

Lattice structures with excellent specific stiffness, specific strength, and energy absorption capability are widely used in the aerospace, transportation, and biomedical industries for load bearing, impact mitigation, and energy absorption [1,2,3,4,5,6]. With the development of additive manufacturing technology, lattice structures with multi-scale and complex cell topology can be fabricated, facilitating designs for special requirements and expanding property modulation within design [7,8]. The cell topologies of lattice structures in nature are usually irregular and the shape of their single cells is random. Therefore, the performance of these structures in load bearing and energy absorption is unsatisfactory. To improve the mechanical properties of lattice structures, many efforts have been made to design structures with periodic and regular cell topologies [9,10,11,12].

Recently, studies on improving the mechanical properties of BCC lattice structures have increased in number. These include structures composed of different density gradient topologies [13,14,15,16,17,18,19], hybrid lattice structures composed of different cell topologies [20,21,22], structures formed by struts with varying cross-sections [23,24,25,26,27,28], structures with arc-shaped struts [29,30], and node-strengthened structures [28,31,32,33]. Chen et al. [23] replaced the struts of a BCC structure with shells. The new structure relieved the stress concentration at the nodes and exhibited high specific energy absorption. For structures with a relative density of ~10%, the relative elastic modulus increased by 2.4 times, and the relative compressive strength increased by 5.4 times compared to a BCC lattice structure. Yang et al. [13] studied BCC structures with different densities and topologies. The study showed that gradient-type BCC structures had stable connections, high modularity and strength at small strains, and layer-by-layer strengthening characteristics at large strains. Bai et al. [29] proposed a curved strut structure with BCC struts in the shape of a circular arc or ellipse. This structure changed the stress distribution of the original structure and effectively relieved the stress concentration at the nodes. Ma et al. [34] proposed a mechanical design strategy, and they applied this method to study BCC lattice structures with an optimized node radius. Sun et al. [35] designed a lattice structure (Y structure) with a negative Poisson’s ratio based on the bionic principle, inspired by grapefruit peel. Meanwhile, the Y structure was mixed with the BCC structure in three ways to simplify the overall lattice structure. Zhao et al. [24] designed BCC structures composed of hollow struts and investigated the mechanical properties with different internal hollow parameters under periodic boundary conditions. When the internal hollow size increased, the elastic modulus of the hollow strut BCC lattice structure increased significantly, and its deformation modes gradually changed from bending-dominated to stretch-dominated. According to Maxwell’s criterion [36], the struts of the lattice structure are usually divided into bending-dominated and stretch-dominated deformations. The bending-dominated structures have a relatively higher specific energy absorption, while the stretch-dominated structures exhibit a higher yield strength because they are directly subjected to tension or compression. Therefore, stretch-dominated lattice structures are mainly used for load bearing in structural applications. In this study, a new lattice structure is proposed by adding a central cube to the BCC structure. 

Because of the limitations of additive manufacturing, 3D-printed lattice structures usually include defects such as cracks and micro-porosity in struts [37,38,39,40,41,42]. Furthermore, printed struts with different tilt angles in the structure can exhibit different mechanical properties [43]. Both of these factors can negatively affect the mechanical performance of the structure. When establishing finite element (FE) models of lattice structures for studying their mechanical properties, the influence of fabrication defects cannot be neglected. Bill et al. [42] pointed out that current computational models for lattice structures based on the FE method often use idealized CAD geometry. The printing defects that commonly occur during additive manufacturing are usually neglected. Such computational models are oversimplified. To overcome this limitation, they incorporated geometric defects (i.e., variation in cross-sectional geometry along the strut length or the ‘waviness’ of the strut) into the struts, and this improved the model’s predictive accuracy. Sun et al. [43] tested the tensile mechanical properties of struts printed in 0° and 45° and assigned these different material properties to struts with varied inclination directions in the lattice structure. The FE model with angle-dependent material properties showed significantly improved predictive accuracy compared to the model with uniform material properties. Alghamdi et al. [44] presented a method to quantify the geometry of as-manufactured lattice structures from microscope images, which showed noticeable deviation from an idealized lattice structure. Based on this approach, the generated FE model could give a good prediction of simulation. Considering the complex topology of lattice structures, a simplification method for modeling lattice structures is discussed in [45]. Amirpour et al. [46] studied the influence of material overlapping (i.e., one type of printing defect) at the nodes on the mechanical properties of polymeric lattice structures. The result showed that the effect of material overlapping was significant for lattices with large-aspect-ratio unit cells. Cao et al. [47] investigated the mechanical response of lattice structures with random geometric defects (i.e., strut porosity, strut thickness variation, and strut corrugation) induced during the additive manufacturing process. The morphology and distribution of 3D printing-induced defects were captured using X-ray computed tomography (XCT) and introduced into the FE model. Better agreement was observed between the predicted results of the FE model and the experimental results.

Using XCT to study the influence of printed defects on the mechanical properties of lattice structures is an efficient and direct method currently available to us [48,49,50,51,52,53]. However, it cannot precisely capture defects in lattice specimens prepared from polymeric materials. In response to the problem of fabrication defects affecting the mechanical properties of polymer lattice structures, this study proposes the incorporation of microcracks observed in the printed specimens into the FE model, along with a material damage model. 

This work proposes a new lattice structure by adding a cubic truss in the center of the BCC structure and aims to improve mechanical properties through a combination of vertical struts (stretch-dominated) and inclined struts (bending-dominated) in a single cell. The optimal size of the central cubic truss is investigated at first. The mechanical properties of the 4 × 4 × 4 lattice structure are studied in detail with an optimally sized central cubic truss. Furthermore, the influence of printing defects on the mechanical properties of the lattice structure is explored numerically by proposing a FE model with incorporation of microcracks at the nodes of the structure.

## 2. Experiments

In this section, a new lattice structure is proposed by adding a cube to BCC structure. The method of fabrication and geometrical parameters of specimens are given. Meanwhile, the experimental procedures and relevant properties are introduced, including energy absorption, bending strength of struts, and deflection of the vertical struts of the central truss.

### 2.1. Design of Lattice Structures

**Figure 1:**
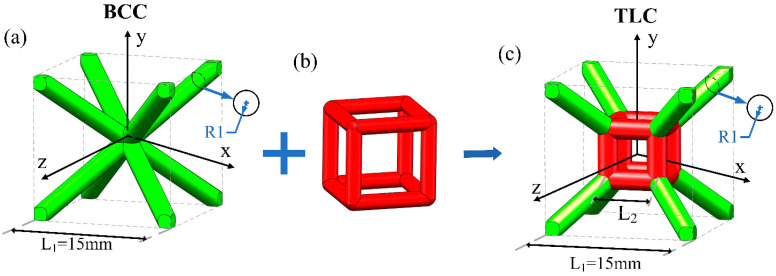
Schematic diagrams of (**a**) BCC cell topology, (**b**) a cubic truss, and (**c**) TLC cell topology (the length of a single cell is 15 mm and the diameter for all struts is 2 mm).

The body-centered cubic (BCC) structure is a typical lattice structure dominated by bending [31], as shown in Figure 1a, which consists of eight struts up and down with equal length and diameter. The BCC lattice structure exhibits an excellent post-yield stress plateau under compressive loading, which enables the structure to possess a high energy absorption efficiency. However, the low bearing capacity of struts in the BCC structure leads to a low stress level, which limits its energy absorption capacity and specific strength. In order to design a new lattice structure possessing a stable stress level similar to the BCC lattice structure and a higher energy absorption capacity, the center node of the BCC lattice is replaced by a cubic truss, as shown in Figure 1b. Four vertical struts are added to the new structure for directly resisting axial compression. The aim of this is to increase the yield strength and flow stress level of the new structure. Meanwhile, the energy absorption capacity can be improved. The proposed structure is denoted TLC (truss–lattice–cube), as shown in Figure 1c. The dimension of a single cell is 15 mm and the diameter of all struts is 2 mm. It is evident that different lengths of the central cubic truss in the TLC lattice structure will exhibit different mechanical properties. The optimal size of the cubic truss is analyzed by experimental tests and numerical simulations, the details of which will be discussed in Section 4.1.

Table 1 shows the key parameters of the BCC and TLC structures. The relative density in Table 1 is the ratio of the lattice volume (obtained from Solidworks (2018) (Dassault Systemes, MA, USA)) to the apparent volume of the lattice structure. Here, the apparent volume refers to the sum of the solid volume and the closed pore volume of the lattice structure. Apparent density is the ratio of the actual mass (obtained from electronic balance, HZK-JA210S (Fuzhou Huazhi, Fuzhou, China)) of the lattice structure to the apparent volume. The theoretical mass of the lattice structures is obtained using Cura 4.8.0 software (along with UltimakerS5 (Ultimaker Holding B.V., Geldermalsen, The Netherlands)).

### 2.2. Additive Manufacturing of Lattice Structures

**Figure 2:**
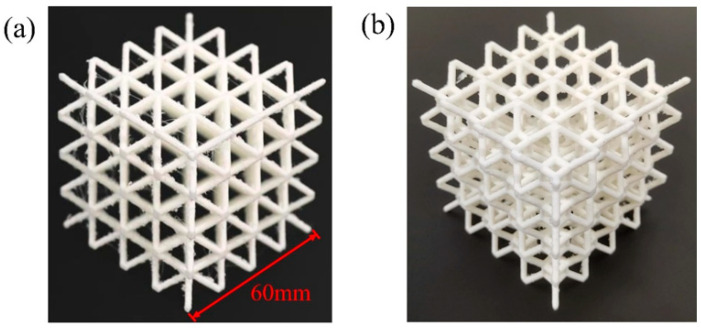
3D-printed specimens of the 4 × 4 × 4 lattice structures: (**a**) BCC and (**b**) TLC.

The 4 × 4 × 4 BCC and TLC lattice structures are built using Solidworks (2018). After that, the lattice models are saved in stereolithography (STL) format and transferred to the Cura software that comes with the UltimakerS5 3D printer for slicing the lattice models. The specimens of BCC and TLC structures printed by the UltimakerS5 3D printer are shown in Figure 2. The printing material for the lattice structures is PolyMax^TM^ PLA with a diameter of 2.85 mm. The UltimakerS5 3D printer has dual printheads: the AA printhead and the BB printhead. In order to print lattice structures with better precision, the BB nozzle is employed to fill the structure with 2.85 mm diameter PolyMax polyvinyl (Polymaker, Changshu, China) alcohol (PVA) water-soluble material. The PVA is used as the substrate in the suspended part of the structure. During the printing of lattice specimens, the temperature of the glass plate is set to 60 °C, the printing layer height is set to 0.1 mm, and the printing temperature is 220 °C. Considering the fabrication quality of the specimens as well as the fabrication time, the nozzle moving speed is set to 55 mm/s, and the pumping distance is 10 mm. The external size of all 4 × 4 × 4 specimens is 60 mm and the diameter of struts is 2 mm.

### 2.3. Mechanical Tests

**Figure 3:**
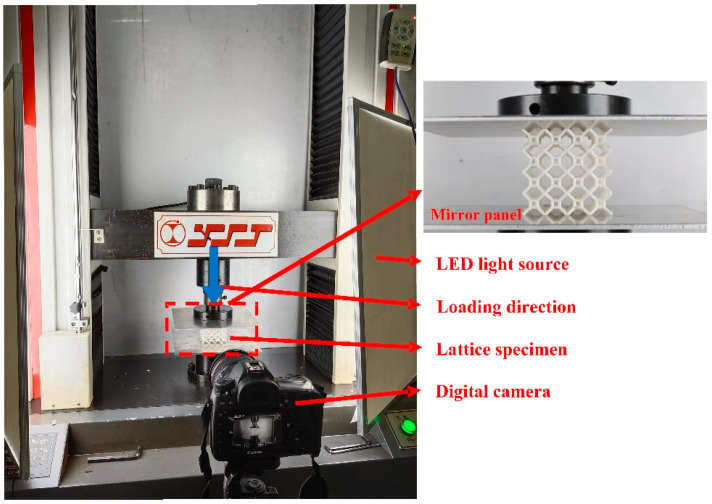
Experimental setup for quasi-static compression tests of lattice structures (the downward blue arrow indicates the direction of compression).

Quasi-static compression tests of the 4 × 4 × 4 lattice structures are performed on an electronic universal tensile and compression testing machine (WDS-100 (Jinan Xinshijin Testing Machine Co., Ltd., Jinan, China.)). To ensure the accuracy of the lattice structures tests, a mirror aluminum alloy plate with a length × width of 200 × 200 mm and a thickness of 5 mm is placed above and below the lattice specimen to reduce the tangential friction. The deformation behavior of each lattice specimen is recorded with a high-resolution digital camera. Backlight plates are placed on the left and right sides of the testing machine to improve the quality of images, as shown in Figure 3. During the test, the upper plate is moved downward to compress the specimen, while the lower plate is fixed. The experimental test continues until the specimen begins to densify (the strain is about 0.75~0.8). The speed of the upper plate is set at 3.6 mm/min, corresponding to a strain rate of 0.001 s^−1^.

To study the optimal size of the central cubic truss in the TLC, single cell and 2 × 2 × 2 lattice specimens are employed for experimental tests. When specimens are compressed to densification, the loading force does not exceed 4 kN. In order to avoid the large error caused by the universal testing machine (WDS-100) with loading force up to 100 kN, a 5 kN universal tensile and compression testing machine (ZQ-990LA (Dongguan Zhi Taking Precision Instrument Co., Dongguan, China)) is used to test the single cell and 2 × 2 × 2 lattice specimens. The compression direction of all specimens in this paper is consistent with the 3D printing direction. Three specimens are tested for each structure. 

### 2.4. Preliminary Analysis

#### 2.4.1. Energy Absorption and Specific Strength

One of the main objectives of present study is to design a new cell topology based on the traditional BCC lattice structure to enhance mechanical properties. The mechanical properties are evaluated based on the energy absorbed (*EA*) per unit volume, the specific energy absorption (*SEA*), the energy absorption efficiency (*EAE*), and the specific strength of the lattice structures.

The *EA* per unit volume of a lattice structure can be obtained by calculating the area enclosed by the stress–strain curve and its corresponding given strain [54,55,56]:

(1) $$EA=\int_{0}^{\varepsilon }\sigma \left(\varepsilon \right)d\varepsilon$$

where *ε* represents the engineering strain and *σ*(*ε*) represents the corresponding engineering stress. Since the BCC and TLC structures have different relative densities, in addition to the EA, the SEA is also considered. *SEA* refers to the energy absorption per unit mass of the lattice structure [54,55,56]:

(2) $$SEA=\frac{\int_{0}^{\varepsilon }\sigma \left(\varepsilon \right)d\varepsilon }{\rho }$$

where *ρ* represents the density of the lattice structure. The structure with higher SEA has a better energy absorption capacity. The *EAE* can be calculated as follows [49]:

(3) $$EAE=\frac{\int_{0}^{\varepsilon }\sigma \left(\varepsilon \right)d\varepsilon }{\sigma (\varepsilon )}$$

This parameter is used to quantify the stability of the post-yield response of the energy absorption. The load-bearing capacity of the lattice structure is evaluated by the specific strength, which refers to the ratio of the corresponding compressive strength under different strains to the apparent density of the lattice structure [57]:

(4) $$\mathrm{SpecificStrength}=\frac{\sigma \left(\varepsilon \right)}{\rho_{A}}$$

(5) $$\rho_{A}=\frac{M}{V_{A}}$$

where $\sigma \left(\varepsilon \right)$ represents the compressive stress, $\rho_{A}$ represents the apparent density of the lattice structure [57], *M* is the mass and *V_A_* is the apparent volume of the lattice structure, which is the solid volume plus the pore volume. It is obvious that the structures with higher specific strength exhibit better load-bearing capacity.

#### 2.4.2. Bending Strength of Struts

The bending strength is the maximum stress that the struts in the lattice structure can withstand when they are under bending loads. It reflects the ability of the material to resist bending and is used to measure the bending performance of the material.

(6) $$\sigma_{\max}=\frac{M_{\max}}{W}$$

(7) $$M_{\max}=\frac{FL}{2}$$

(8) $$W=\frac{\pi d^{3}}{32}$$

where $M_{\max}$ represents the maximum bending moment of the strut, *W* represents the bending section factor of the strut, *F* represents the external force applied, and *L* represents the lever arm. As all struts of the lattice structure in this study have the same diameter, the flexural section factor is fixed, i.e., W=0.785.

#### 2.4.3. Deflection of Vertical Central Struts

When symmetry of applied force on single cells is broken due to damage or fracture at any location of the lattice structure, lateral deflection of vertical struts will happen, and this will reduce the load-carrying capacity of the structure. In this study, the deflection $y_{\max}$ is used as a variable to evaluate the optimal size of the central cubic truss of the TLC lattice structure. When the lattice structure bears the same load, the larger the deflection, the more likely its vertical struts are to be eccentric, resulting in more frequent damage to the lattice structure. The deflection formula is shown as follows:

(9) $$y_{\max}=-\frac{F_{x}L_{2}^{3}}{3EI}$$

(10) $$I=\frac{\pi d^{4}}{64}$$

where $F_{x}$ represents the lateral force applied to the vertical struts, *L*_2_ represents the length of the cubic truss at the center of the TLC structure, *EI* represents the bending rigidity, and *I* represents the second moment of area. Since the diameter of struts and the elastic modulus of the material are the same, the factor affecting strut deflection is only related to the length of the central cubic truss, *L*_2_. Hence, the selection of the length *L*_2_, the size of the central cubic truss, is crucial to its mechanical properties.

## 3. Finite Element Modeling

In this section, the printing material properties are obtained by testing dog-bone specimens. A detailed description of the finite element model and numerical simulation, including boundary conditions, element types, loading and meshing, is given.

### 3.1. Material Properties

**Figure 4:**
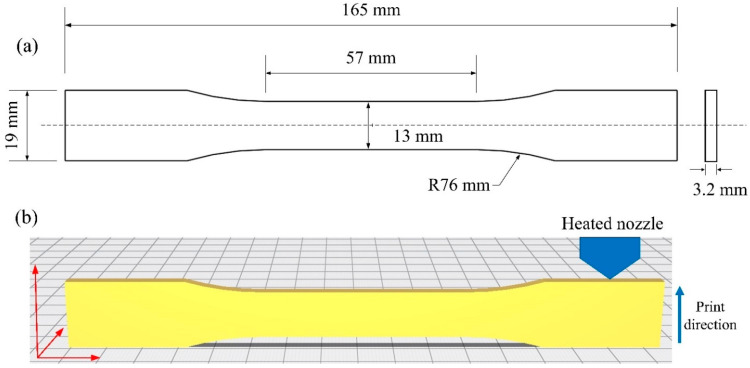
(**a**) Dimensions of ASTM D638 Type I dog-bone specimens; (**b**) 3D image of ASTM D638 Type I dog-bone specimen for printing (the blue arrow represents the direction of FDM-based 3D printing).

**Figure 5:**
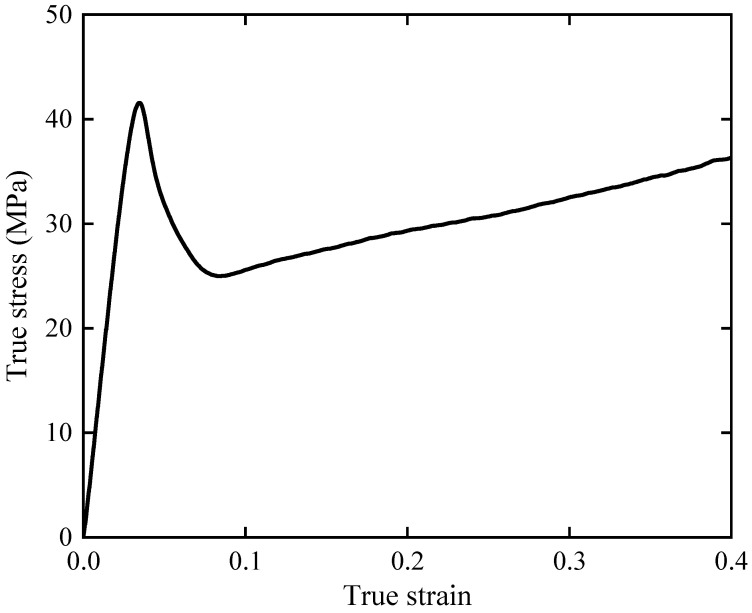
True stress–true strain curve of PLA.

When the lattice structure is subjected to compression, stresses in different struts are either tensile or compressive. For polymer materials used in this study, the stress resulting in tensile failure is much smaller than the stress resulting in compressive failure. Therefore, as a simplified approach, tensile properties of the printing material are adopted as the material parameters used in FE modeling [14,54,55]. ASTM D638 [58] Type I dog-bone specimens are fabricated using the same manufacturing parameters as the lattice structure to test the mechanical properties of PolyMax^TM^ PLA materials [54]. In Figure 4a, the dimensions of the ASTM D638 Type I dog-bone specimens are given. Figure 4b shows the 3D image of the dog-bone specimen for printing. The tensile properties of three dog-bone specimens are tested at 3.41 mm/min using the universal testing machine (ZQ-990LA). The true stress–strain curve is shown in Figure 5. The elastic and plastic material parameters are given in Table 2 and Table 3, respectively.

### 3.2. Finite Element Model

**Figure 6:**
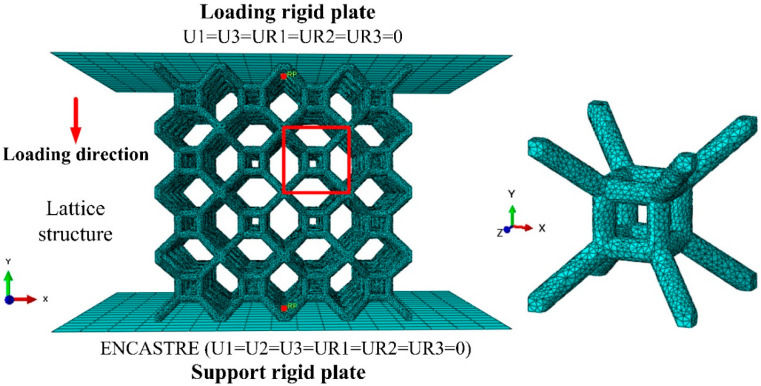
Finite element model with boundary conditions and the red square defining a single cell of TLC structure meshed with C3D4 tetrahedral elements.

The FE model of a 4 × 4 × 4 lattice structure is established for uniaxial compression in ABAQUS 6.14. Figure 6 shows the FE model of the TLC lattice structure. There are three parts in the numerical model: the loading plate, the lattice structure, and the support plate. The loading and support plates adopt a three-dimensional discrete rigid shell plane. All the lattice models use C3D4 tetrahedral solid elements and the mesh size is set to 0.65 mm. The support plate fixes all degrees of freedom, and the loading plate only retains the degrees of freedom in the “y” direction. The loading plate is set to a downward displacement of 48 mm with a speed of 3.6 mm/min, which is consistent with the experimental strain rate of 0.001 s^−1^. In the simulation, the large deformation of lattice structure induces complex self-contact between structural struts. The contact between the loading plate and the lattice structure adopts “general contact”. The friction formula for the tangential behavior is “penalty”, and the friction coefficient is set to be 0.2 [59]. For FE simulations of complex structures, especially for nonlinear compression with large deformations, it is usually calculated using ABAQUS/Explicit [60,61]. Compared to ABAQUS/Standard, explicit solvers allow for reductions in computational resources and time and also avoid convergence problems that may be encountered with implicit solvers. To further reduce the computational time of the simulation, the mass scaling function of ABAQUS is used. Keeping the ratio of kinetic energy to internal energy below 5% and ensuring that the artificially introduced inertial effects are minimized, the simulation can be considered quasi-static compression [60,61]. Based on many attempts, the time period is set to 0.01 on the premise of ensuring the accuracy of the simulation results.

## 4. Results and Discussion

In this section, the optimal size of the central cubic truss of the TLC lattice structure is investigated.

The mechanical properties and deformation modes of the TLC and BCC lattice structure are discussed in detail. The influence of manufacturing defects (i.e., microcracks) on the mechanical properties of the lattice structure is studied in detail.

### 4.1. The Optimal Size of the Central Cubic Truss in the TLC Structure

**Figure 7:**
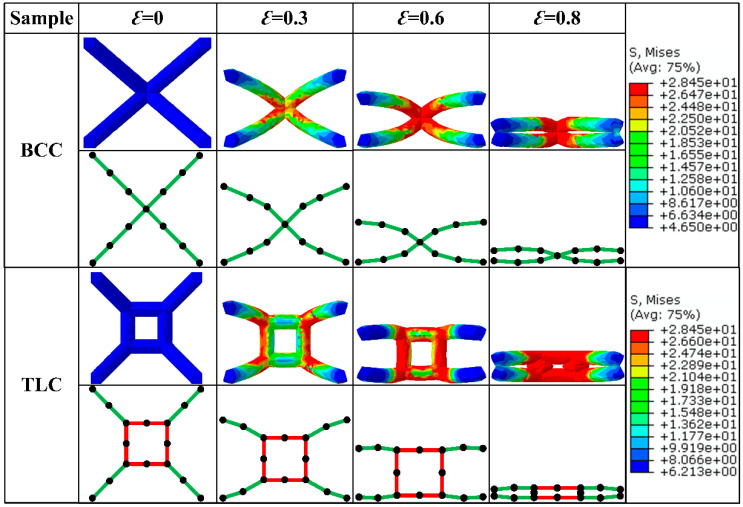
Deformation characteristics of BCC and TLC single-cell lattice structures with different strains.

Figure 7 shows the deformation characteristics of single cells for the BCC and TLC lattice structures at engineering strains of 0, 0.3, 0.6, and 0.8. For the BCC lattice structure, the eight struts are deformed mainly by bending. With the increase in strain, deformation of bending proceeds until the strain reaches 0.8, at which point the upper strut comes into contact with the lower strut. Thereafter, the structure starts to densify. In contrast, the deformation of the TLC lattice structure includes two stages: bending for eight inclined struts and compression for the vertical struts of the central cube. 

**Figure 8:**
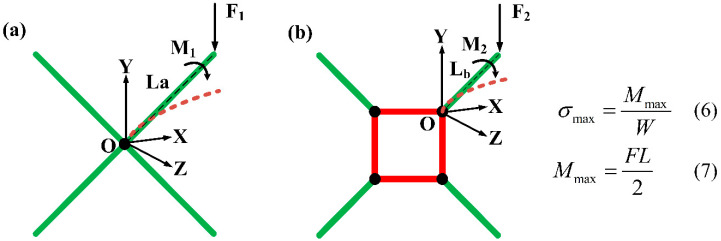
Schematic diagram of force analysis of inclined struts in (**a**) the BCC lattice structure and (**b**) the TLC lattice structure.

When the inclined strut is subjected to a vertical load and the diameter of the strut is fixed, the length (L) of the strut determines its flexural strength, as indicated by Equations (6) and (7) in Figure 8. It is evident that the shorter the strut, the greater the bending strength. For lattice structures in this study, the bending strength of inclined struts directly affects the yield strength. The shorter inclined struts in the TLC structure endow it with higher yield strength.

**Figure 9:**
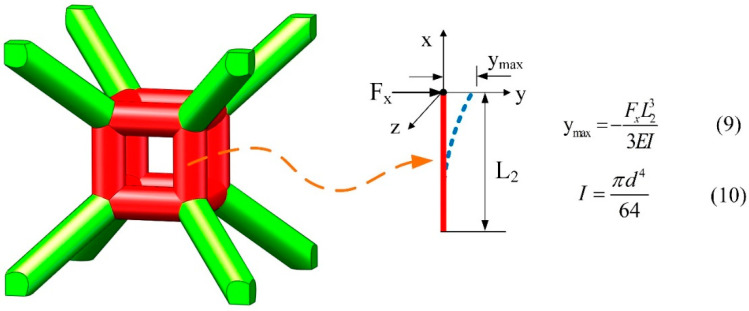
Deflection of the vertical strut in the center of the TLC lattice structure due to lateral force (assuming that the lower end of the vertical strut is fixed).

With respect to the buckling of columns, the vertical struts of the central truss belong to short columns, and Euler’s formula is not applicable. Figure 9 is a schematic diagram of the deflection of the central cubic strut due to lateral force. When the TLC structure is under compression, premature damage or fracturing may happen in some struts due to printing defects. Therefore, the symmetry of applied force on single cells may be broken and the vertical struts of the central truss will be subjected to a resolved lateral force alongside the axial loading. Lateral deflection would then be unavoidable and the whole structure would be prone to instability. As shown in Figure 9 with Equations (9) and (10), when the diameter of the strut is the same, the longer the length *L*_2_ of the veritical strut, the more easily deflection will occur, which leads to the destruction of the lattice structure at lower strains.

**Figure 10:**
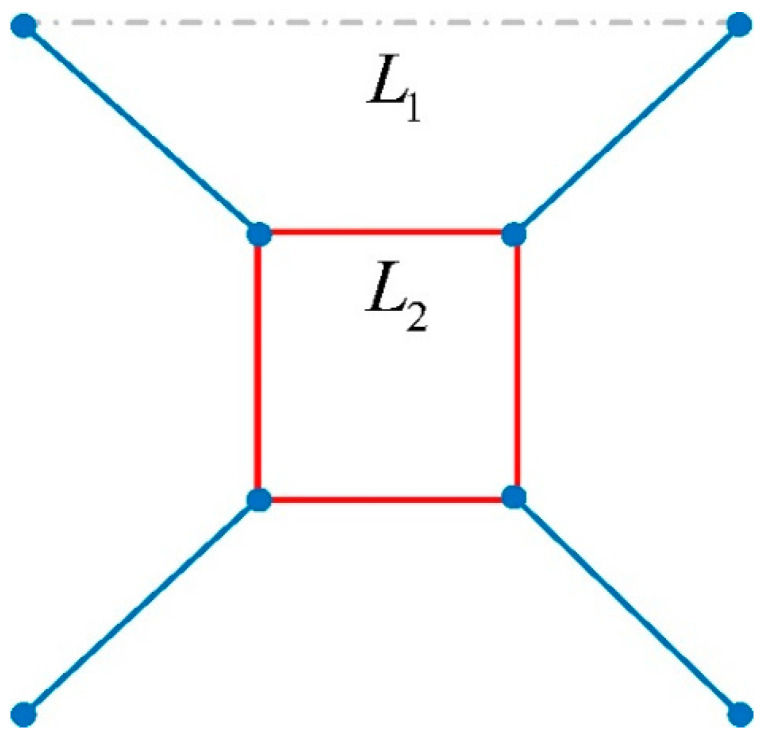
Schematic diagram of the cell topology of the TLC lattice structure.

Figure 10 shows the schematic diagram of the cell topology of the TLC lattice structure, where *L*_2_ represents the length of the cubic truss and *L*_1_ the size of the single cell. *L*_1_ has a fixed value of 15 mm for all structures. The optimal length of *L*_2_ is investigated in this study. *L*_2_ takes values of 3 mm, 4 mm, 5 mm, 6 mm, 7 mm, and 7.5 mm, respectively, as shown in Table 4. The TLC structures with six values of central cubic trusses are analyzed by experimental tests and numerical simulations in the following.

**Figure 11:**
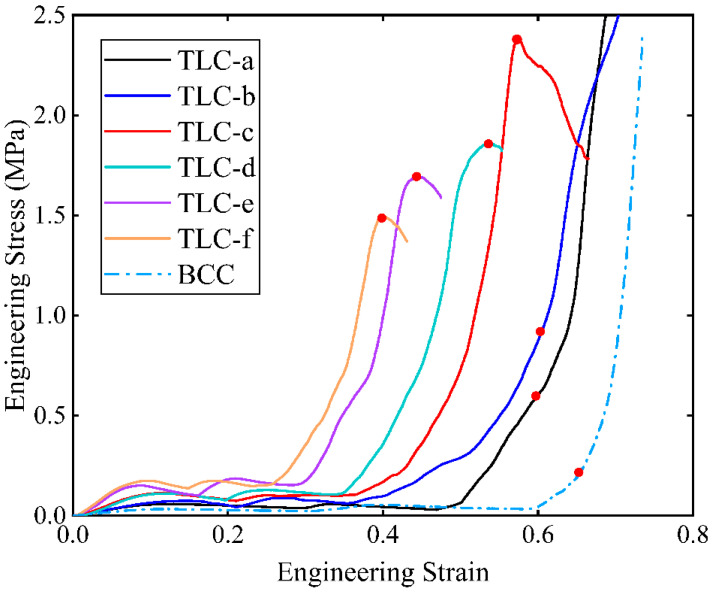
Compressive stress–strain curves of single cells obtained from experiments (a–f denote different lengths of the central cubic truss, as given in Table 4).

**Figure 12:**
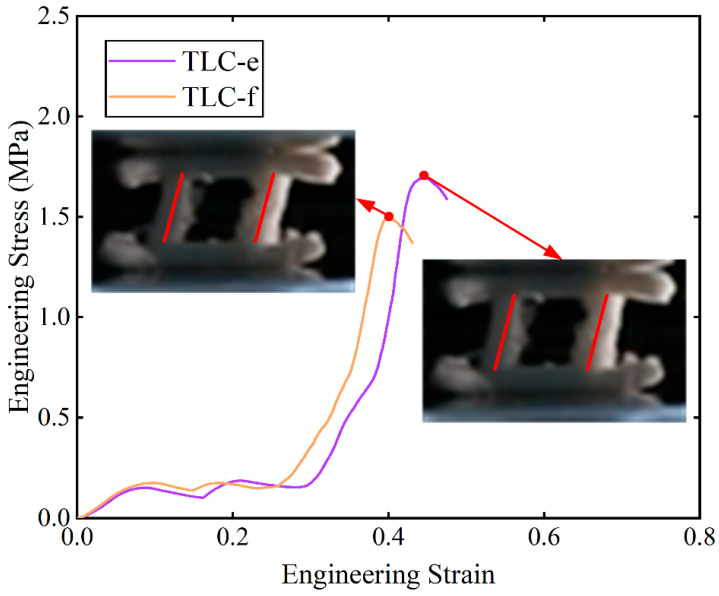
Compressive stress–strain curves for TLC-e and TLC-f; the insets show the deformed specimens at the highest stress points.

Figure 11 shows the engineering stress–strain curves obtained from compression tests for single cells. The red points marked on the curves indicate the highest stresses of the structure during compression and before densification. Among these curves, the stresses of the TLC-e and TLC-f begin to decrease at a strain of ~0.4. Figure 12 shows the deformation characteristics of the TLC-e and TLC-f structures at their highest stresses. It is consistent with above analysis: the longer the vertical strut (*L*_2_) is, the earlier deflection happens. Furthermore, this deflection results in earlier destruction of the single cell. Therefore, TLC-e and TLC-f are not the best choices for the central truss. In contrast, *L*_2_ of TLC-a is the shortest and the deflection of the vertical struts is more difficult. However, the increased length of the inclined struts will cause decreased yield strength. As shown in Figure 11, the stress–strain curve of TLC-a almost coincides with that of the BCC structure, and its mechanical properties are not obviously improved compared to the BCC structure. Similarly, the stress level of the TLC-b structure is not significantly elevated compared to the BCC structure. Therefore, both TLC-a and TLC-b should not be regarded as the optimal sizes for the TLC structure. 

**Figure 13:**
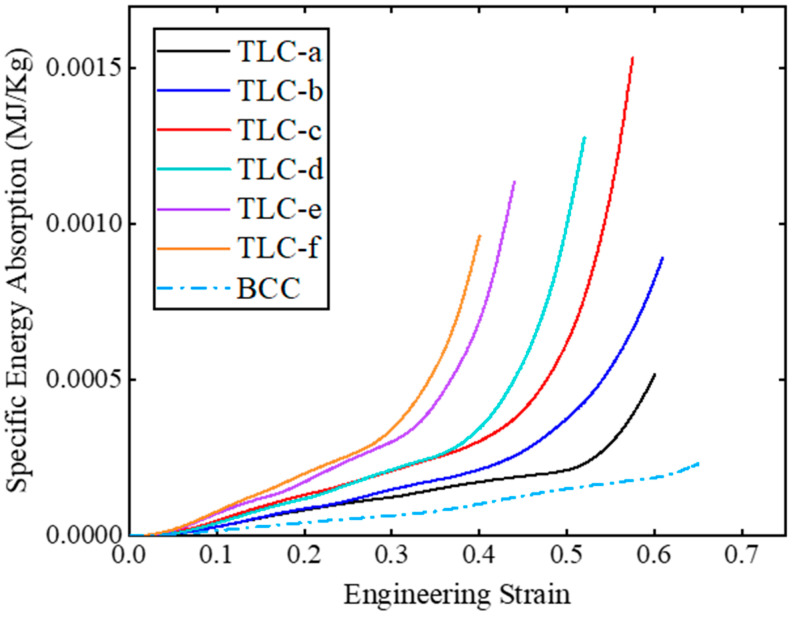
Specific energy absorption corresponding to various strains for TLC and BCC single cells.

**Figure 14:**
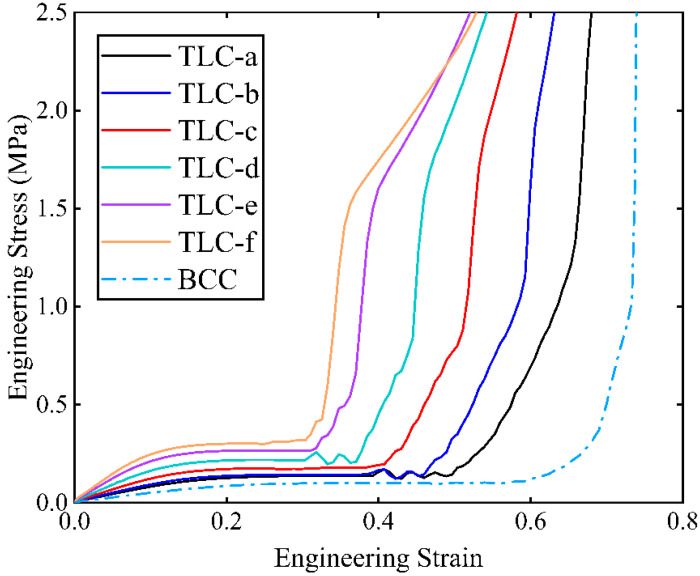
Simulated compressive stress–strain curves of single cells of the TLC and BCC structure.

Figure 13 shows the *SEA* curves of TLC and BCC single cells with respect to strains. Among the six sizes, TLC-c (i.e., the red curve) has the best energy absorption capacity. However, before the strain reaches 0.5, the performance of the TLC-d structure is equal to or even better than that of TLC-c. The simulation results in Figure 14 clearly illustrate the higher flow stress level of TLC-d compared to that of TLC-c. Therefore, observing only the behavior of single cells, it is difficult to determine which of the two values is the optimal size for the central cubic truss in the TLC structure.

**Figure 15:**
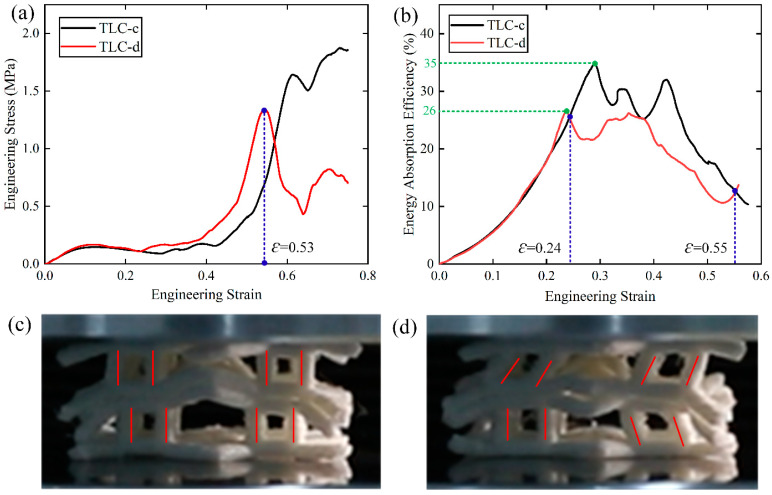
(**a**) Engineering stress–strain curves of the 2 × 2 × 2 TLC-c and TLC-d obtained from experimental tests; (**b**) energy absorption efficiency curves (the green dashed lines represent the highest energy absorption); (**c**,**d**) deformation characteristics of TLC-c and TLC-d at a strain of 0.53, respectively.

In order to explore the mechanical properties of TLC-c and TLC-d in-depth, 2 × 2 × 2 specimens of TLC-c and TLC-d are fabricated for experimental tests, and the results are shown in Figure 15. The blue dashed line in Figure 15a indicates the strain corresponding to the highest stress in TLC-d. It shows that before a strain of 0.53, the stress level of TLC-d is a little higher than that of TLC-c. Figure 15b shows the energy absorption efficiency (*EAE*) curves of 2 × 2 × 2 TLC-c and TLC-d structures. The maximum *EAE* values of the TLC-c and TLC-d structures are 35% and 26%, respectively. TLC-c exhibits a 1.35 times higher *EAE* than TLC-d. Between strains from 0.24 to 0.55, the *EAE* of TLC-c is obviously superior to that of TLC-d. Figure 15c,d show the deformation characteristics of TLC-c and TLC-d at a strain of 0.53, respectively. It is apparent that the vertical struts in TLC-d have been significantly laterally deflected, and the whole structure is unstable. In contrast, the vertical struts in TLC-c can still resist the axial load properly. That is to say, although the flow stress of TLC-d is a little higher than that of TLC-c, the TLC-c structure exhibits more stability than TLC-d. Particularly for large-scale lattice structures or under dynamic loading, the high stability of the structure can generate a high energy absorption capacity. Therefore, considering three factors together—i.e., the *SEA*, stability of the structure, and the flow stress level—TLC-c exhibits the best performance. That is to say, the optimal length of the central cubic truss *L*_2_ is determined to be 5 mm. Therefore, the size of the central truss is fixed at 5 mm in the following study. 

### 4.2. Experimental Results for 4 × 4 × 4 Structures

**Figure 16:**
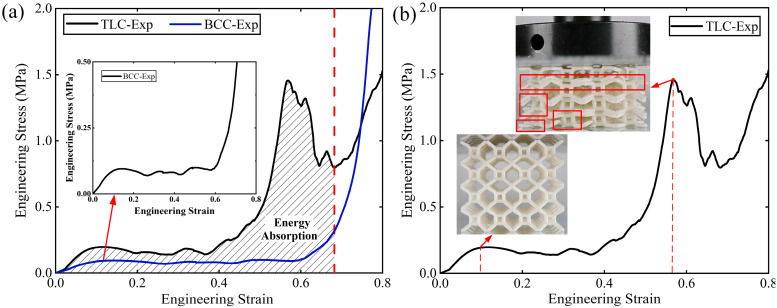
(**a**) Engineering stress–strain curves of 4 × 4 × 4 BCC and TLC; (**b**) deformation characteristics of TLC specimens with different strains.

The mechanical properties and energy absorption of lattice structures in this study are discussed based on the results of quasi-static compression tests. The engineering stress–strain curves of 4 × 4 × 4 lattice structures are shown in Figure 16a. From the figure, it can be found that the deformation in the BCC and TLC lattice structures generally occurs in three stages. The first stage is elastic deformation; the stress rises elastically with strain until it reaches the yield strength. After this point, the struts undergoing bending start to yield plastically. The curve enters the second stage, and there is a plateau with a small fluctuation in stress. With the increase in strain, the curves of stress enter the third stage; the flow stress of structures continues to increase. For example, when the strain of the TLC lattice structure reaches 0.4, after exhausting bending deformation in eight tilted struts in a cell, axial deformation begins to dominate, which is mainly contributed by the vertical struts of the central cube. Thereafter, the stress will increase significantly. However, when the strain reaches about 0.6, the stress of the TLC structure decreases abruptly. The reasons for this will be discussed later in this section. The deformation characteristics of TLC structures at strains for initial yielding and the highest stress before densification are shown in Figure 16b. The red squares mark the points at which struts break during compression.

As shown in Figure 16a, the flow stresses of the TLC structure are much higher than those of the BCC structure. For instance, the initial flow stress of the TLC structure is increased by 122% compared to that of the BCC structure at a strain of 0.1. The stress of the TLC at a strain of 0.4 is increased by 191%. When the strain increases from 0.5 to ~0.6, the stress of the TLC structure rises to its highest point, 1.45 MPa, while the corresponding stress of the BCC is only 0.1 MPa. The vertical struts of the central cube resist the axial loads, and the energy absorption capacity of this structure is improved accordingly. The shaded part under the engineering stress–strain curve is the energy absorbed by the TLC lattice structure. The value is much higher than that absorbed by the BCC lattice structure, which indicates that the energy absorption capacity of the TLC lattice structure is significantly improved.

**Figure 17:**
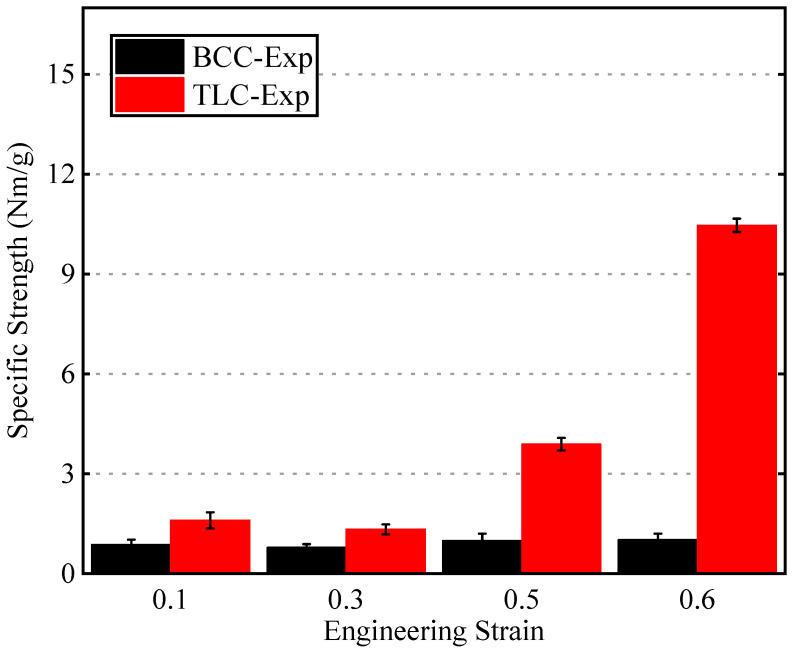
Specific strength of the BCC and TLC lattice structures at different strains.

Figure 17 shows specific strength values corresponding to the BCC and TLC at strains of 0.1, 0.3, 0.5, and 0.6, respectively. The BCC structure shows lower specific strength compared to the TLC structure, and its values exhibit minimal changes with strain. The proposed TLC lattice structure shows much better specific strength, indicating an improved load-bearing capacity. For instance, at a strain of 0.5, the specific strength of the TLC is increased by 293% compared to that of the BCC structure.

**Figure 18:**
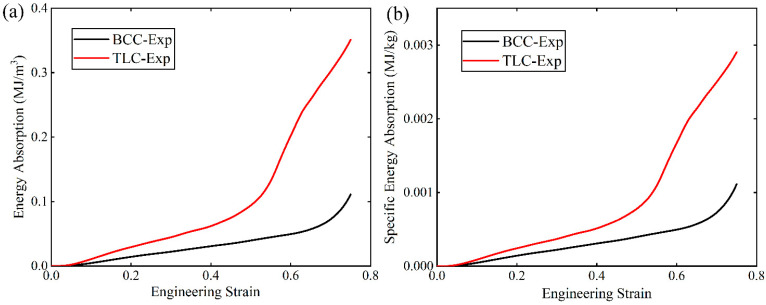
(**a**) Energy absorption and (**b**) specific energy absorption of BCC and TLC lattice structures with strain.

Figure 18 shows *EA* and *SEA* of the BCC and TLC lattice structures. The performance of the TLC is much better than the BCC structure. At strains of 0.5 and 0.6, the *EA* of the TLC is enhanced by 96% and 181%, respectively, and the *SEA* is enhanced by 136% and 312%, respectively, compared to that of the BCC structure.

**Figure 19:**
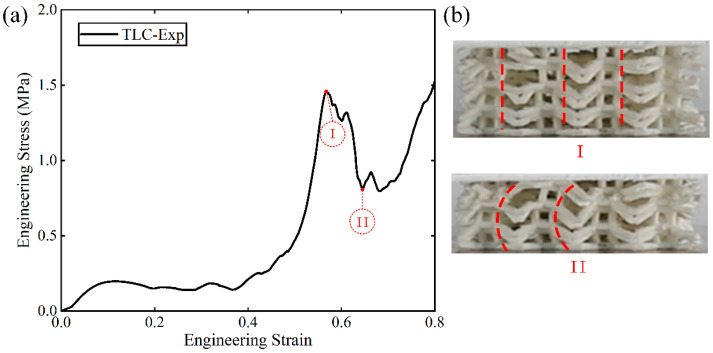
(**a**) The stress–strain curve of the 4 × 4 × 4 TLC structure; (**b**) images of TLC specimens at points I and II (the dashed red lines in the images indicate the status of the central vertical struts).

A salient point is worth further discussion: as shown in Figure 19a, the curves for the TLC structure exhibit crests at strains just before the final densification of the structure. In contrast, for the BCC structure, such a crest in the stress–strain curve cannot be observed. Figure 19b shows the corresponding images of the TLC specimens at strains near the crest and the trough. Image I denotes the crest and Image II the trough. It is obvious that in Image I, the vertical struts of four layers up and down the central cube are almost in a straight line, as indicated by the dashed red lines. They work together like one vertical column to resist compressive loads. When compression proceeds, the vertical struts of four layers cannot stay in a straight line anymore, as shown by the dashed red lines in Image II. Lateral deflection or damage may happen to these vertical struts. Thereafter, destruction of the whole structures continues. Therefore, the crests and the trough near densification in the TLC structure are found to be the results of the deformation of vertical struts in the central cubic truss.

### 4.3. Finite Element Modeling for Lattice Structures with Defects

**Figure 20:**
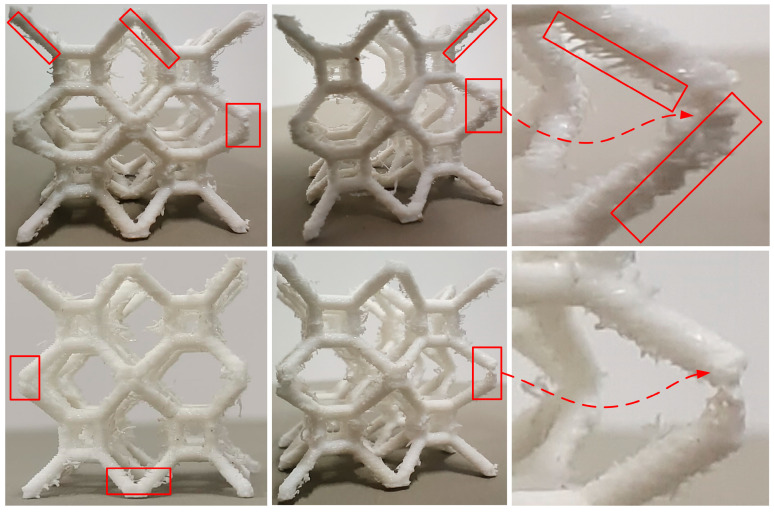
3D printing defects in 2 × 2 × 2 specimens of TLC lattice structures.

While 3D printing technology makes the fabrication of complex structures possible, defects induced by printing are inevitable. Figure 20 shows 2 × 2 × 2 specimens of the TLC lattice structure printed using FDM technology. The red rectangles highlight the places where printing defects are obvious. These images clearly show two typical categories of defects during the printing of polymer lattice structures: pores and microcracks in the struts or nodes/joints. Although these defects may be small, they can have a significant negative impact on the mechanical properties of lattice structures; thus, they cannot be ignored. Specifically, pores and microcracks in the inclined struts can clearly be observed in Figure 20, and the weak connection between the two inclined struts can easily be found. These printing defects could result in premature damage to or fracture of the struts, and the mechanical performance of the whole structure will deteriorate significantly. 

**Figure 21:**
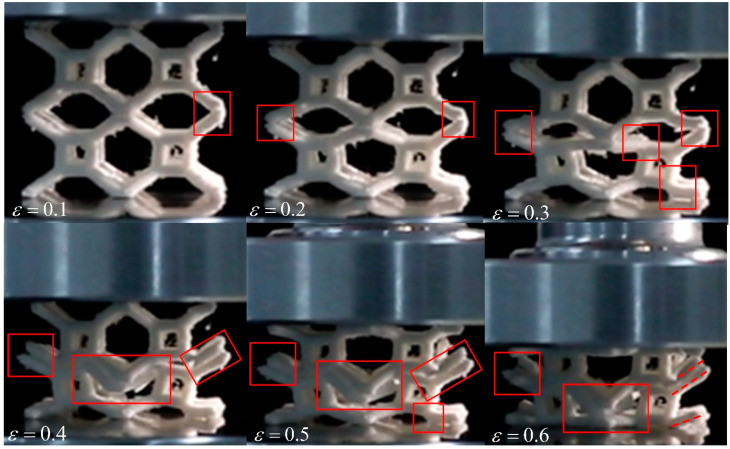
Deformation characteristics of 2 × 2 × 2 TLC lattice structures in compressive tests.

Figure 21 shows the deformation characteristics of 2 × 2 × 2 TLC specimens at different strains. In the initial phase of the compressive deformation—i.e., a strain of 0.1, it can be found that the node highlighted by the red rectangle shows an obvious crack. Similar cracks can be found in nodes at a strain of 0.2. When the strain reaches 0.3, serval nodes and struts show clear fracturing, and the uniform deformation of the lattice structure can therefore not be maintained anymore. As compression proceeds (i.e., at strains of 0.4, 0.5 and 0.6), a collapse occurs the middle layer, which is supported by inclined struts; all inclined struts break and lose their function.

**Figure 22:**
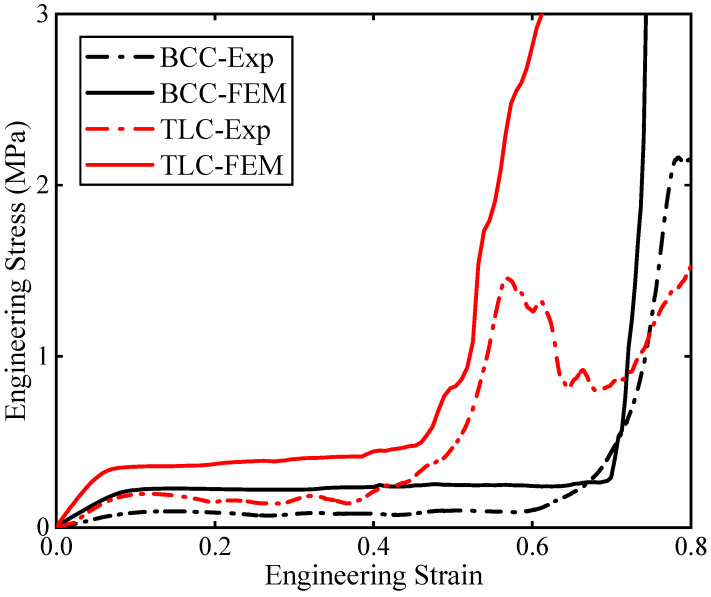
Engineering stress–strain curves of experimental and simulation results for BCC and TLC 4 × 4 × 4 lattice structures.

Figure 22 shows the experimental and simulation results for both 4 × 4 × 4 BCC and TLC lattice structures. The solid lines are for experiments and the dashed lines for simulations. Although the trend for both results is consistent, the flow stresses exhibit obvious discrepancy between them. The key point is that the FE model adopts an ideal lattice structure while 3D printing defects in experimental structures cannot be avoided. With close observation of the experimental specimens, microcracks in printed struts often lead to uneven stress distribution and local damage when the specimens are subjected to compressive loads. Such asymmetric structural stresses could result in unexpected lateral resolved forces, which may further cause the overall collapse and failure of the lattice specimens. However, in the numerical simulation, the model is usually simplified: the struts are designed to ignore printed defects and the nodes of the lattice structure are assumed to be perfectly connected. With the increase in strain, the damage and fracture of the struts in the lattice specimens grow gradually for experiments; in contrast, the ideal structure in the numerical simulation can always maintain a continuous and uniform deformation. Therefore, it is necessary to consider the influence of printed defects during FE modeling for lattice structures to improve the accuracy of the numerical simulation. This work proposes the incorporation of microcracks into the FE model for the TLC lattice structure. Due to high requirements in terms of computational resources after insertion of microcracks in the FE model, only 2 × 2 × 2 lattice structures are considered in the following simulations.

#### 4.3.1. Establishment of the Finite Element Model for Lattice Structures with Microcracks

**Figure 23:**
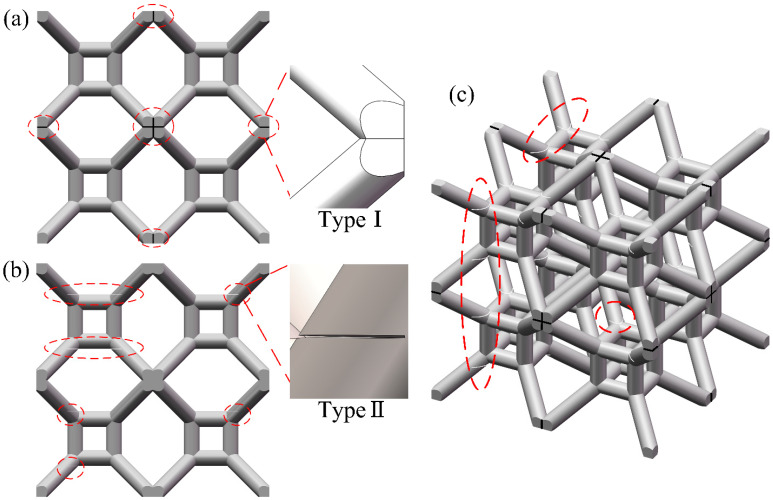
TLC lattice structure model with microcracks located at (**a**) Type I nodes; (**b**) Type II nodes; and (**c**) both Type I and II nodes.

It is worth noting that defects in lattice specimens printed by FDM technology are random. The number and location of defects in each lattice specimen are uncertain, making it challenging to establish a FE model with similar defects for a lattice specimen. Based on experimental observation, two salient points can be concluded: (i) inclined struts in lattice specimens often have more defects than vertical struts; (ii) nodes are the locations of stress concentration, and defects at or near nodes can have a higher impact on mechanical properties than defects at other places. Considering the feasibility of FE modeling, it is proposed in this study that microcracks are explicitly inserted into the lattice structure at or near nodes to capture the influence of printing defects on mechanical performance. In this study, nodes in the TLC lattice structure are categorized into two types: (i) nodes between inclined struts (referred to as Type I nodes), and (ii) nodes between inclined struts and vertical struts (referred to as Type II nodes). The two types of nodes have different effects on mechanical properties and will be discussed later. For Type I nodes, all printing defects on inclined struts and/or on nodes are represented by an equivalent microcrack located at the center of the Type I node (i.e., the struts are disconnected but in full contact), as shown in Figure 23a. For Type II nodes, an equivalent microcrack will be inserted at one end of the strut near the Type II node (i.e., cracks with 50% width of the strut and 0.02 mm thickness), as shown in Figure 23b. 

In order to study the influence of defects at different locations on the overall mechanical properties of the structure, three FE models are established for TLC lattice structures: Model A (the model with only microcracks at the Type I nodes), Model B (the model with only microcracks at the Type II nodes), and Model C (the model with microcracks at both types of nodes).

There are 19 Type I nodes and 64 Type II nodes in a 2 × 2 × 2 TLC specimen. Based on experimental observation, Type I nodes usually exhibit weak connection. Therefore, microcracks are inserted into all the Type I nodes, except the one at the center of the lattice structure, as shown in Figure 23a. Since there are a large number of Type II nodes, all the Type II nodes being weakened by printing defects is not realistic. As a primary estimation, 32 nodes (i.e., half of all the Type II nodes) were randomly selected to add microcracks, as shown in Figure 23b. Figure 23c shows the lattice structure with inserted microcracks located at both Type I and II nodes, in which the number and location of microcracks are consistent with the previous two structures.

In this study, the built-in ductile damage model in ABAQUS is employed for the three models, and the localized failure is simulated through element deletion. The material parameters are determined based on comparison with the experimental results of 2 × 2 × 2 lattice specimens. The fracture strain in in the ductile damage model is set to 0.12 and the fracture energy is set to 2. The other material parameters are set to be consistent with those given in Section 3.

#### 4.3.2. Simulation Results 

**Figure 24:**
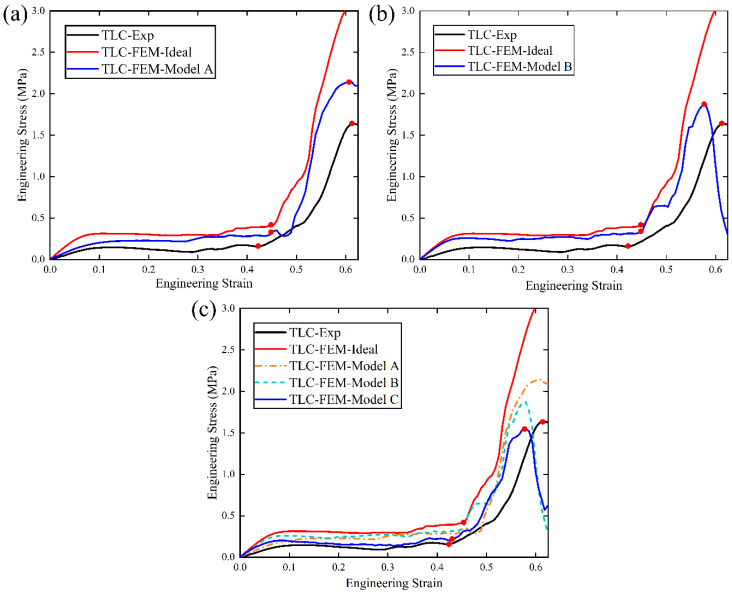
(**a**) Comparison of stress–strain curves between simulations of ideal models, Model A, and experiments; (**b**) comparison of stress–strain curves between simulations of ideal models, Model B, and experiments; (**c**) comparison of stress–strain curves between simulations of ideal models, Model C, and experiments (the dashed lines for Model A and B are added in (**c**) for reference).

Figure 24a–c show the compressive simulation results for the three models: Model A, B, and C, denoted by blue curves. The black curve represents the experimental result and the red curve is the simulation result for a 2 × 2 × 2 ideal lattice structure. Several interesting points can be noted from the results: (1) It is obvious that the trends of the three blue curves in Figure 24a–c from the three models are consistent with our experimental results (indicated by the black curve). The red dots highlight the transition points and the maximum stress points on the curves. In contrast, the red curve for the ideal lattice structure does not show a maximum stress, and its flow stress continues to increase as compression proceeds. This is because the ideal structure does not consider the damage of the material. (2) The influences of microcracks at Type I and Type II nodes on the mechanical properties of the structures are different and can be easily observed from the results for Model A and B in Figure 24. Compared to the ideal lattice structure, Figure 24a shows that microcracks inserted at Type I nodes can significantly weaken the initial flow stress. Specifically, at a strain of 0.1, the initial flow stress decreases significantly compared to that of the ideal structure, and it is only 43% higher than that of the experimental results; meanwhile, the stress of the ideal structure is 121% larger than that of experiments (see Table 5). In Figure 24b, microcracks inserted at Type II nodes cause the maximum flow stress to decrease significantly compared to that of Model A, and the maximum flow stress only 14% higher than that of the experimental result. However, its influence on the initial flow stress is not as strong as that of the microcracks at Type I nodes. (3) The simulation results from Model C in Figure 24c exhibit excellent agreement with the experimental results, particularly compared with results from Models A and B (see Table 5). That is to say, the FE model including both types of microcracks can generate much better predictions.

**Figure 25:**
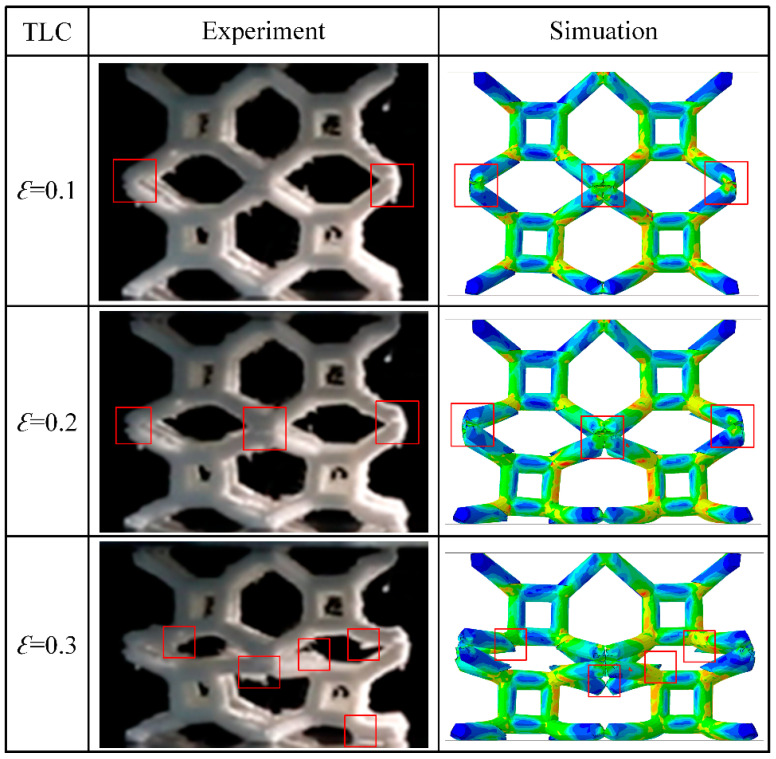

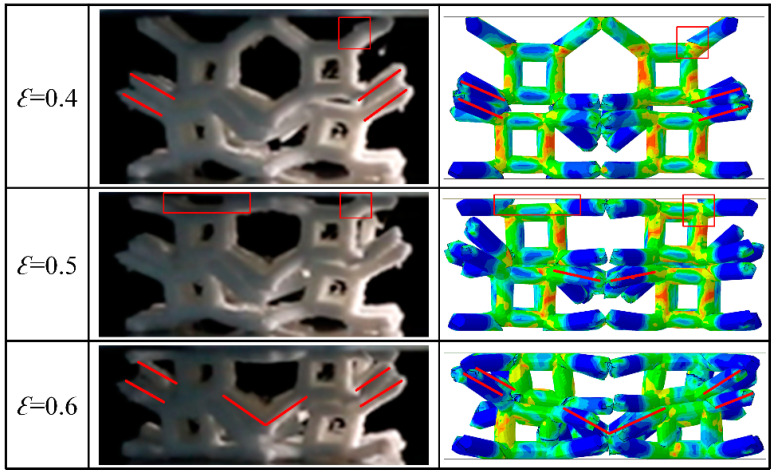
Comparison of deformation characteristics between experiments and simulations of Model C at various strains (The red boxes and lines highlight the locations with similar characteristics of damage/failure between experiments and simulations).

Figure 25 shows the deformation characteristics of a 2 × 2 × 2 lattice structure for experiments and FE simulations of Model C. The damage and fracturing of struts in experimental specimens can easily be observed with an increase in strain. Such an evolution of damage and fracture in the lattice structure is captured very well in the simulation of Model C, which includes microcracks at both Type I and Type II nodes. At a strain of ~0.1, damage is usually initiated at Type I nodes, where there are connection points of two inclined struts. When the compressive strain increases to 0.2, cracks at Type I nodes can easily be observed, as highlighted by the red rectangle in the figure, although the structure remains intact as a whole. This indicates that the Type I nodes are the location at which premature damage can occur easily, especially in the presence of printing defects. The simulations exhibit inhomogeneous deformations similar to those in our experiments, and the internal structure appears tilted as well. As the load increases, complete fracturing at Type I nodes as well as damage at Type II nodes can be clearly observed in both experiments and simulations, as marked by the red boxes in the image showing a strain of 0.3. This will lead to further damage of the whole structure. With continued deformation, the middle layer of the lattice structure appears to collapse. The inclined struts related to the middle layer are totally fractured at a strain of 0.4. As the strain increases, the other inclined struts are fractured gradually, and vertical struts are also heavily deformed when strain reaches 0.6.

Based on the above simulation results, it is evident that presence of the printing defects (i.e., microcracks in this study) significantly affects the mechanical properties of the structure. Incorporation of microcracks into the lattice structure at both Type I and II nodes, as proposed in this work, could help the FE simulation to capture the deformation characteristics of lattice structures very well and significantly improve the predictive accuracy of simulations.

## 5. Conclusions

Based on the BCC lattice structure, this study proposes a new structure, TLC, by adding a central cubic truss into the BCC structure. The combination of vertical struts (stretch-dominated) and inclined struts (bending-dominated) in a single cell is demonstrated to significantly improve the mechanical properties of the TLC in terms of initial flow stress, specific strength, and specific energy absorption. 

The optimal size of the central cubic truss in the TLC is studied numerically and experimentally. With a fixed unit cell size (15 mm) and strut diameter (2 mm), the optimal size for the central cube is determined to be 5 mm, considering the factors of flow stress, specific energy absorption, and the stability of the structure. A general finding is that shorter inclined struts will generate a higher yield stress while corresponding longer vertical struts will make the whole structure unstable at a lower strain. 

The experimental results show that the mechanical properties can be significantly improved by adding a central cubic truss of an optimal size into the BCC lattice structure. For a 4 × 4 × 4 TLC structure, the initial flow stress of the TLC structure is increased by 122% at a strain of 0.1; the specific strength is enhanced by 293% at a strain of 0.5; and the specific energy absorption is increased by 312% at a strain of 0.6, compared to that of the BCC structure.

This study proposes the incorporation of microcracks into an FE model of a TLC lattice structure, and the simulations capture the influence of printing defects on mechanical properties very well. Microcracks at Type I nodes can notably decrease the initial flow stress of the TLC, while microcracks at Type II nodes can lower the maximum stress of the structure to a remarkable degree. The FE model featuring microcracks at both Type I and Type II nodes makes simulations with good predictive accuracy compared to the experimental results. The simulations from this model exhibit excellent consistency with the deformation characteristics of the TLC from experiments, including damage and fractures in the structure at small strains.

## Figures and Tables

**Table 1:** Geometrical parameters of the PolyMax^TM^ (Polymaker, Changshu, China) PLA lattice structures.

Structure	Volume	Theoretical Mass	Actual Mass	Relative Density	Apparent Density
BCC	18.37 cm^3^	21.70 g	21.55 g	0.0850	0.0998
TLC	22.11 cm^3^	26.09 g	26.11 g	0.1024	0.1209

**Table 2:** The elastic material parameters of PolyMax^TM^ PLA.

Elastic Modulus (GPa)	Poisson’s Ratio	Density (kg/m^3^)	Yield Strength (MPa)
1.85	0.35	1200	42

**Table 3:** The plastic material parameters of PolyMax^TM^ PLA.

Plastic strain	0	0.0045	0.012	0.021	0.034	0.132	0.2	0.3
Plastic stress (MPa)	40	36	32	29	26	28	30	34

**Table 4:** Length of the central cubic struts.

$L_{2}/L_{1}$	3/15 a	4/15 b	5/15 c	6/15 d	7/15 e	7.5/15 f
$L_{1}$	15 mm	15 mm	15 mm	15 mm	15 mm	15 mm
$L_{2}$	3 mm	4 mm	5 mm	6 mm	7 mm	7.5 mm

**Table 5:** Comparison of flow stresses between experimental and simulation results (MPa).

	Initial Flow Stress at a Strain of 0.1	Maximum Stress
Experiment	0.14	1.64
Ideal structure	0.31(121% ↑)	3.00 (83% ↑)
Model A	0.20 (43% ↑)	2.15 (31% ↑)
Model B	0.26 (86% ↑)	1.87 (14% ↑)
Model C	0.19 (36% ↑)	1.55 (6% ↓)

## Data Availability

Data are contained within the article.
